# Regeneration of Transected Recurrent Laryngeal Nerve Using Hybrid-Transplantation of Skeletal Muscle-Derived Stem Cells and Bioabsorbable Scaffold

**DOI:** 10.3390/jcm7090276

**Published:** 2018-09-12

**Authors:** Akihito Kazuno, Daisuke Maki, Ippei Yamato, Nobuyuki Nakajima, Hiroya Seta, Shuichi Soeda, Soji Ozawa, Yoshiyasu Uchiyama, Tetsuro Tamaki

**Affiliations:** 1 Department of Gastroenterological Surgery, Tokai University School of Medicine, 143 Shimokasuya, Isehara, Kanagawa 259-1193, Japan; tokaikazuno@yahoo.co.jp (A.K.); sozawa@tokai.ac.jp (S.O.); 2 Muscle Physiology & Cell Biology Unit, Tokai University School of Medicine, 143 Shimokasuya, Isehara, Kanagawa 259-1193, Japan; d.maki@tokai.ac.jp (D.K.); ippei-y@is.icc.u-tokai.ac.jp (I.Y.); nakaji.n@is.icc.u-tokai.ac.jp (N.N.); hrystx0168@gmail.com (H.S.); shu10206@kaname-clinic.com (S.S.); y-uchi@is.icc.u-tokai.ac.jp (Y.U.); 3 Department of Otolaryngology, Tokai University School of Medicine, 143 Shimokasuya, Isehara, Kanagawa 259-1193, Japan; 4 Department of Medical Education, Tokai University School of Medicine, 143 Shimokasuya, Isehara, Kanagawa 259-1193, Japan; 5 Department of Urology, Tokai University School of Medicine, 143 Shimokasuya, Isehara, Kanagawa 259-1193, Japan; 6 Department of Orthopedics, Tokai University School of Medicine, 143 Shimokasuya, Isehara, Kanagawa 259-1193, Japan; 7 Department of Human Structure and Function, Tokai University School of Medicine, 143 Shimokasuya, Isehara, Kanagawa 259-1193, Japan

**Keywords:** vocal cord function, p75, N200, qPCR, immunohistochemistry

## Abstract

Hybrid transplantation of skeletal muscle-derived multipotent stem cells (Sk-MSCs) and bioabsorbable polyglyconate (PGA) felt was studied as a novel regeneration therapy for the transected recurrent laryngeal nerve (RLN). Sk-MSCs were isolated from green fluorescence protein transgenic mice and then expanded and transplanted with PGA felt for the hybrid transplantation (HY group) into the RLN transected mouse model. Transplantation of culture medium (M group) and PGA + medium (PGA group) were examined as controls. After eight weeks, trans-oral video laryngoscopy demonstrated 80% recovery of spontaneous vocal-fold movement during breathing in the HY group, whereas the M and PGA groups showed wholly no recoveries. The Sk-MSCs showed active engraftment confined to the damaged RLN portion, representing favorable prevention of cell diffusion on PGA, with an enhanced expression of nerve growth factor mRNAs. Axonal re-connection in the HY group was confirmed by histological serial sections. Immunohistochemical analysis revealed the differentiation of Sk-MSCs into Schwann cells and perineurial/endoneurial cells and axonal growth supportive of perineurium/endoneurium. The number of axons recovered was over 86%. These results showed that the stem cell and cytokine delivery system using hybrid transplantation of Sk-MSCs/PGA-felt is a potentially practical and useful approach for the recovery of transected RLN.

## 1. Introduction

The recurrent laryngeal nerve (RLN) comprises two non-symmetrical branches of the vagus nerve looping under the aortic arch (left-side) and the right subclavian artery (right-side), both traveling upwards alongside the trachea. The role of the RLN is control of all intrinsic muscles of the larynx (except for the cricothyroid muscle), and carrying of sensory information from the mucous membranes of the larynx below the lower surface of the vocal cord, as well as the sensory, secretory, and motor fibers to the cervical segments of the esophagus and the trachea. Therefore, when the RLN is damaged and/or transected unexpectedly through surgery (thyroid gland, lung, esophagus, mediastinum, and heart) or accident, recurrent nerve paralysis is induced and accompanied by paralysis of the vocal-fold movement (unilateral), resulting in a raspy voice, breathlessness during conversation, and lack of voice as typical side-effects. Currently, there is no effective treatment, and natural healing is not expected in human patients, impacting them functionally, physiologically, and emotionally. This results in associated stress and depression, thus causing impaired quality of life. Similar insufficient natural recovery of transected RLN is also observed in rodent models [1,2]. The nerve autograft is the current gold standard therapy in this case. However, the sacrifice of a healthy nerve graft is necessary, and this should be avoided as much as possible. The RLN runs through quite a narrow interspace between the trachea and esophagus, and is affected by various pressure changes, unavoidably produced by the movement of the trachea and esophagus. Capillary action with surface tension in this narrow space also fosters the diffusion of substances, making treatment difficult. 

Several methods to regenerate transected RLN have been utilized. The drug delivery system using a scaffolding biomaterial and neurotrophic factors, such as brain-derived neurotrophic factor (BDNF) and glial cell line-derived neurotrophic factor (GDNF), was proposed instead of the autologous nerve graft, and showed neuro-functional EMG improvement [3]. Direct injection of cultured rat CD56^+^ myoblasts into the thyroarytenoid muscle was also performed, expecting ciliary-derived neurotrophic factor (CNTF) expression, and their paracrine factors affected the neighboring transected RLN indirectly and improved laryngeal electromyography [4]. Bone marrow mesenchymal stem cells were also applied intravenously in the rat RLN crash injury model and showed favorable functional recovery of vocal fold motion; however, direct contribution of these stem cells to nerve regeneration was unclear [5]. Therefore, the results are not direct proof of morphological regeneration of the RLN and functional recovery of vocal-fold movement; thus, there is still room for methodological improvement. 

We have proposed and demonstrated the significant therapeutic capacity of skeletal muscle-derived multipotent stem cell (Sk-MSC) and human skeletal muscle-derived stem cell (Sk-SC) transplantation to the transected sciatic nerve, showing high numerical and functional recovery [6,7]. The regenerative capacity of mouse Sk-MSCs/human Sk-SCs (i.e., differentiation into Schwann cells, perineurial/endoneurial cells, vascular pericytes, endothelial cells, and smooth muscle cells) has been demonstrated in various tissue circumstances such as in the sciatic nerve [6,7], damaged skeletal muscle [8,9,10], facial nerve network [11], urethral sphincter [12,13], bladder wall [14], and the nerve-vascular bundle [15]. Furthermore, we recently developed a hybrid-transplantation method for Sk-MSCs and bioabsorbable polyglyconate (PGA) felt and achieved significant innervation and vascularization in the bronchial stump [16]. The benefit of PGA tube usage on transected RLN regeneration was also reported using the rat model [17]. Therefore, the purpose of this study was establishment of a novel method to obtain the morphological and functional regeneration of gap-transected RLN using hybrid-transplantation of the mouse Sk-MSCs and PGA felt.

## 2. Experimental Section

### 2.1. Animals

Green fluorescent protein transgenic mice (GFP-Tg mice; C57BL/6 TgN[act EGFP]Osb Y01; provided by Dr. M. Okabe, Osaka University, Osaka, Japan) [18] were used as donor mice for cell transplantation (age 4–8 weeks, *n* = 12), and wild-type mice (C57BL/6N) were used as recipients (age 8–12 weeks, *n* = 33). All experimental procedures were approved by the Tokai University School of Medicine Committee on Animal Care and Use (No. 175011), and we also confirmed that all experiments were performed in accordance with relevant guidelines and regulations. 

### 2.2. Cell Purification

Muscle sampling was performed under an overdose of pentobarbital (60 mg/kg, Schering-Plough, combined with butorphanol tartrate 2 mg/kg, Meiji Seika, Tokyo, Japan, i.p.). The thigh and lower leg muscles (tibialis anterior, extensor digitorum longus, soleus, plantaris, gastrocnemius, and quadriceps femoris) of GFP-Tg mice were removed and used in subsequent experiments. The average total muscle mass removed was 525 ± 83 mg/GFP-Tg mouse (mean ± SE). Note that the muscles were not minced, and whole muscles were each treated with 0.1% collagenase type IA (Sigma-Aldrich, Tokyo, Japan) in Dulbecco’s modified Eagle’s medium (DMEM) containing 7.5% FCS with gentle agitation for 2 h at 37 °C. After digestion, isolated cells were filtered through 70-, 40-, and 20-μm nylon filters to remove other debris. After washing, cells were cultured in Iscove’s modified Dulbecco’s medium (IMDM) containing 20% FCS (100 units/mL penicillin G, 100 μg/mL streptomycin sulfate, and 10 μg/mL gentamycin sulfate and 0.1 mM β-mercaptoethanol) for 5 days. Expanded cells were gently detached from culture dishes with 2 mM EDTA solution and were impregnated into the PGA felt around the RLN transected area, as described in the next section. Basically, in the above method, skeletal muscle interstitial cells were mainly isolated, and could be divided into the CD34^+^/45^−^ (Sk-34) and CD34^−^/45^−^ (Sk-DN) fractions after fresh isolation [19,20]. In the present study, however, we used a non-sorted mixed cell population after expansion because the therapeutic purpose can be obtained from effective cell expansion. Therefore, the present Sk-MSCs were considered to be mainly obtained by expanding Sk-34 and Sk-DN cells, which are in the same cell lineage in the mouse [21].

### 2.3. Preparation of RLN Transected Model and Application of Hybrid-Transplantation

**Figure 1:**
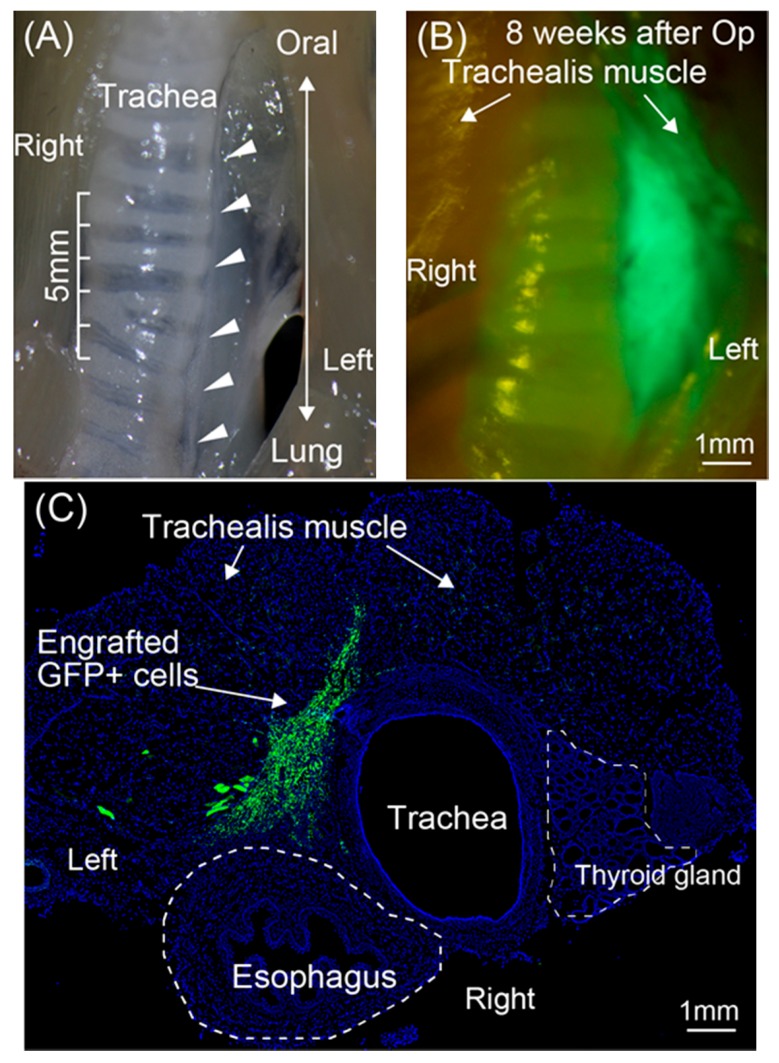
Macroscopic view of recurrent laryngeal nerve (RLN) and macroscopic and histological view of the transplanted Sk-MSCs. (**A**) Macroscopic view of the left RLN and its scale. The RLN runs closely along the trachea (arrowheads). (**B**) Macroscopic view of the engrafted Sk-MSCs transplanted as a hybrid sheet at eight weeks after operation. Green fluorescent protein (GFP^+^) tissues were maintained beside the trachea. (**C**) Histological view of the engrafted skeletal muscle-derived multipotent stem cells (Sk-MSCs) at eight weeks after operation. GFP^+^ cells were also located beside the trachea and esophagus. Blue staining = nuclear staining with 4′,6-diamidino-2-phenylindole (DAPI). Bars = 1 mm.

To obtain the experimental RLN transected model, the trachea was isolated after skin incision and blunt dissection of neck muscles. With retraction of skin and muscles, the left-side RLN was detected under surgical stereomicroscope, and a 5-mm nerve was removed to make a gap (refer to Figure 1A,B). The nerve gap was then covered with PGA felt (0.2–0.4 mg piece), and medium containing expanded Sk-MSCs (5 µL with 1 × 10^5^ cells/µL) was impregnated into the PGA felt. Transplanted hybrid felt was covered with the placed-back neck muscles, and the skin was sutured. 

All surgical procedures were performed under inhalation anesthesia (Isoflurane; Abbott, Tokyo, Japan). After the operation, a transparent sterile/analgesic plastic dressing (Nobecutan spray; Yoshitomi Chemical, Fukuoka, Japan) was sprayed over the wound, and penicillin (4000 units/100 mL) was administered subcutaneously to prevent infection. Basically, three conditions, the hybrid (HY, *n* = 21) group, non-cell media + solo PGA felt (PGA, *n* = 6) group, and non-cell media transplanted control (M, *n* = 6) group were prepared and compared. Additional normal controls were also prepared and analyzed, if necessary. 

### 2.4. Measurement of Vocal Cord Movement 

At 2, 4, and 8 weeks following surgery, under inhalation anesthesia as described above, trans-oral video laryngoscopy was performed to record vocal-fold movement during spontaneous breathing, using a 3.9-mm rigid fine endoscope (Inspection Camera RoHS CE LED, Sanko, Tokyo, Japan). One-time video recording was limited to a 15-s maximum and repeated 3–4 times with a minimal 1-min interval to avoid death from suffocation and maintain anesthesia. The animals were returned to the inhalation state during every interval. The movement score was used to evaluate vocal cord functional recovery based on maximal adduction and abduction on the contralateral intact side (right-side). Scoring was performed using each video clip within the 0–3 point scale (with 0 = no movement (complete paralysis), 1 = slight movement less than 50% of that of the contralateral right side, 2 = moderate movement 50–80% of that of the right side, and 3 = well-moved over 80% of that of the right side), averaged per animal at each stage, and then evaluated as a group.

### 2.5. Macroscopic Observation and Immunostaining 

At 8 weeks after transplantation, recipient mice were given an overdose of pentobarbital (60 mg/kg, +xylazine HCl 10 mg/kg, i.p.), and engraftment of donor-derived GFP^+^ cells in the damaged portion was confirmed by fluorescence stereomicroscopy (SZX12; Olympus, Tokyo, Japan and refer to Figure 1B). Recipient mice were then perfused with warm 0.01 M phosphate buffered saline (PBS) through the left ventricle, followed by fixation with 4% paraformaldehyde/0.1 M phosphate buffer (4% PFA/PB), and samples were removed and fixed overnight in 4% PFA/PB at 4 °C and washed with a graded sucrose (0–25%)/0.01 M PBS series. Samples were then immersed in OCT compound and frozen/stored at −80 °C. Subsequently, 7-µm histological sections were obtained. Localization of nerve fibers (axons) was detected by rabbit polyclonal anti-Neurofilament 200 (N-200, dilution = 1:1000; incubation = room temperature 25 °C for 1 h; Sigma, St. Louis, MO, USA). Schwann cells were detected by anti-p75 (rabbit polyclonal, 1:400, 4 °C overnight; CST, Boston, MA, USA). Myelin formation was detected using rabbit polyclonal anti-myelin basic protein (MBP; 1:200, for 2 h at room temperature; Millipore, Billerica, MA, USA), and rabbit anti-GLUT-1 (1:100, room temperature for 1 h; Diagnostic BioSystems, Pleasanton, CA, USA) was used for identification of the perineurium. Blood vessels were detected with rat anti-mouse CD31 (1:500, 4 °C overnight; BD Pharmingen, San Diego, CA, USA) monoclonal antibody, known as vascular endothelial cell marker. Reactions were visualized using Alexa Fluor-594-conjugated goat anti-rabbit and anti-rat antibodies (1:500, room temperature for 2 h; Molecular Probes, Eugene, OR, USA). Nuclei were counter-stained with DAPI (4,6-diamino-2-phenylindole). Morphological and numerical recovery of RLN was accomplished by a count of the number of anti-N200^+^ axons from the pre-, central-, and post-portion of the defect nerve area. 

### 2.6. Quantitative Real-Time PCR for Peripheral Nerve-Related Growth Factors

To confirm the paracrine effects of Sk-MSCs in vivo after transplantation, expression levels of three mouse nerve growth factors, brain-derived neurotrophic factor (BDNF), glial cell line-derived neurotrophic factor (GDNF), and ciliary neurotrophic factor (CNTF), were determined by using quantitative real-time PCR. For this purpose, mice in HY (*n* = 4) and PGA (*n* = 4) groups were given an overdose of pentobarbital (60 mg/kg, +xylazine HCl 10 mg/kg, i.p.), and engrafted GFP^+^ cells/tissues with PGA (refer to Figure 1B) and solo PGA with little tissue adhering to the trachea were carefully removed under fluorescence stereomicroscopy at 2 weeks after operation. At this time point, the remaining PGA felt was still easily detectable macroscopically. Then, both samples were prepared for real-time quantitative PCR analysis. Total RNA was purified using a QIAGEN RNeasy micro kit (QIAGEN K.K. Tokyo, Japan), according to the manufacturer’s instructions. First-strand cDNA synthesis was performed with the ReverTra Ace qPCR RT Master Mix with gDNA Remover (TOYOBO, Osaka, Japan). Primers were as follows: BDNF [22], CNTF (forward: 5′-CCTTTCCAGGTGTTTAACAGTCTT-3′, reverse: 5′-TCAGTCTTTTCAGAAGTCAACCA-3′), and GDNF [23]. Quantitative PCR was performed using StepOnePlus™ (Applied Biosystems, Foster City, CA) and Taqman Fast Universal PCR Master Mix (2×) No AmpErase UHG (Thermo Fisher Scientific K.K., Tokyo, Japan). The PCR cycle consisted of an initial denaturation step at 95 °C for 20 s, followed by 40 cycles of 95 °C for 1 s and 62 °C for 20 s. The expression was normalized to the level of β-actin [22] and Ribosomal Protein L (RpL) 13a (forward: 5′-ATCCCTCCACCCTATGACAA-3′, reverse: 5′-GCCCCAGGTAAGCAAACTT-3′) mRNA. The gene expression levels were compared between the PGA and HY groups.

## 3. Results

### 3.1. Engraftment of GFP^+^ Cells/Tissue around the RLN

Engraftment of transplanted GFP^+^ Sk-MSCs was firstly confirmed by fluorescence stereomicroscopy (Figure 1B). The GFP^+^ cells/tissue closely adhered to the trachea, which was the area of the RLN transected (Figure 1A,B). This feature was also detected in the histological cross-sections (Figure 1C), and this enabled us to re-confirm the locations of the trachea, esophagus, trachealis muscles, and engrafted GFP^+^ Sk-MSCs, provided by the hybrid transplantation of Sk-MSCs and PGA. 

### 3.2. Functional Recovery of Vocal Cord Movement

**Figure 2:**
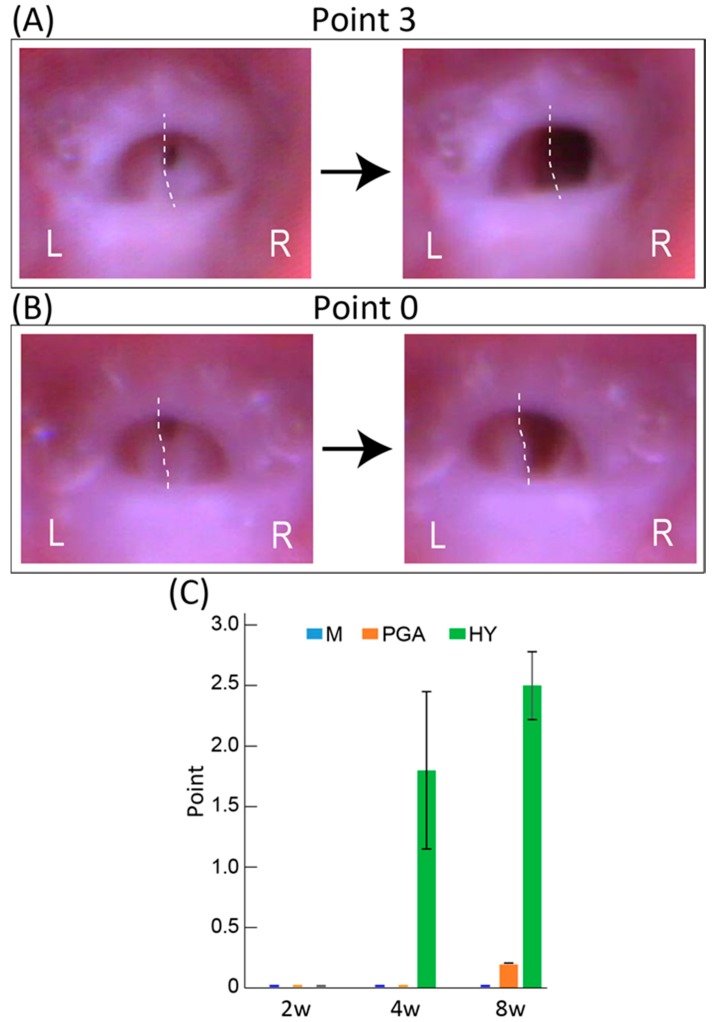
Typical photographs of vocal cord and evaluation of functional recovery of vocal-fold movement. (**A**) Typical photograph of vocal cord with a score of three points. (**B**) Typical photograph of vocal cord with a score of zero points. L, left side; R, right side. Dotted lines show a central situation of the left side (operated side) vocal cord. (**C**) Comparison of the functional recovery of vocal cord movement in the three groups. M, media transplanted group; PGA, PGA transplanted group; HY, hybrid transplantation (Sk-MSCs + PGA) group. The media group showed no recovery, and the PGA group showed very little recovery only at eight weeks. In contrast, the HY group showed gradual and favorable recoveries 4–8 weeks after operation. Note that the video clip of typical vocal-fold movement with the indicated 0–3 grade is available in the Supplementary Materials.

Functional recovery of the RLN was evaluated by the vocal-fold movement detected by trans-oral laryngoscopy (Figure 2A,B) and rating using a 0–3-point scale. Typically, the M group had 0 points, as there was no recovery of vocal cord movement at any stage (Figure 2C). Therefore, the present mouse RLN transected model is considered the irreversible model. The PGA group showed little recovery at eight weeks after transplantation (average 0.3 ± 0.2, Figure 2C). In contrast, the HY group showed over 50% recovery (average 1.8 ± 0.7 points) at four weeks, and the score was further increased to approximately 80% compared with the movement of the contralateral intact side (average 2.5 ± 0.3 points) at eight weeks, whereas there was no movement detected at two weeks (Figure 2C). This gradual functional recovery is also shown in Table 1, indicating the changes in the distribution ratio of the recovery point criteria of the vocal cord in the HY group. The percentages of higher scores were increased following recovery. A video clip of typical vocal-fold movement, rated from 0–3 points, is available in the Supplementary Materials.

### 3.3. Morphological Regeneration of the RLN Demonstrated Using Serial Histological Sections

**Figure 3:**
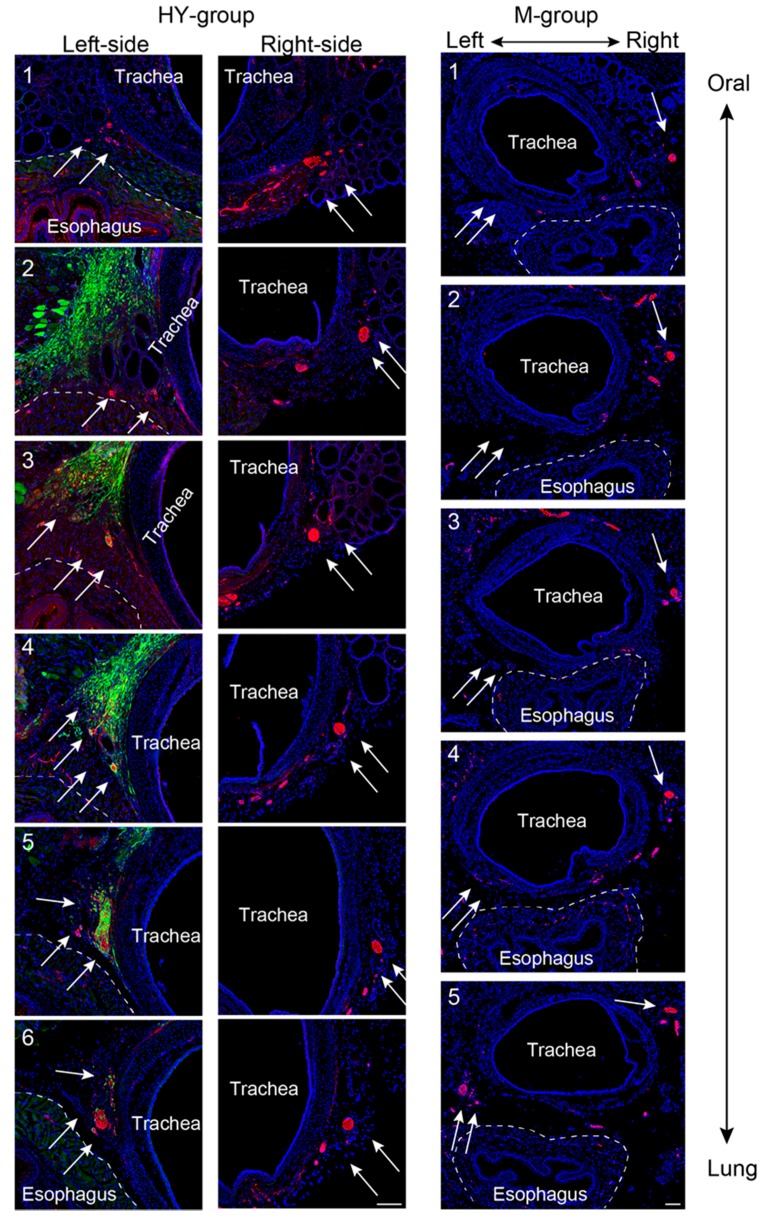
Serial cross-sectional examination of the RLN regeneration in the HY- and M-group. Numbers in each photograph are in serial order from the proximal (oral) to distal (lung) side. Similarly, arrows indicate the expected situation of the RLN both on the right and left side. All sections were stained with anti-N200 as axon staining (red reactions). Axons can be seen through the serial sections of the HY-group both in the contralateral right- and operated left-side. In addition, Sections 3–6 on the left-side of the HY group show encircling of GFP^+^ cells/tissues of N200^+^ axons. However, axons are undetected on the left-side of the M-group, showing the incomplete regeneration of the RLN. Dotted lines show the outermost layer of the esophagus. Blue staining is nuclear staining with DAPI. Bars = 100 μm. The formation with very few axons in the RLN defected portion of the PGA group is shown in Figure S1.

Morphological regeneration of the transected RLN using serial sections from the oral to the lung side (1–6 sections) in the HY and M group is shown in Figure 3. In this figure, location of the RLN can be reconfirmed in the delta regions between the trachea and esophagus, as expected from the macroscopic view as shown in Figure 1A (and also in Figure 3 arrows on left- and right-side of the HY (Panel 6) and M groups (Panel 5)). These data strongly indicated that the left and right RLN were located in the normal left and right delta regions. The GFP^+^ cells were also located close to the left-side of the trachea in the HY group (Figure 3, left-side, arrows). Importantly, axons could not be detected in Panels 1–4 in the M group, while they could be seen in Panel 5 (arrows on left, M group), indicating that the transected RLN did not regenerate. However, in the HY group, axons were observed continuously in the left-side Panels 1–6, showing that the transected RLN was regenerated. In addition, GFP^+^ cells frequently encircled N200^+^ axons (arrows on left-side, HY group), indicating the relationship of regenerated axons and transplanted Sk-MSCs. In addition, the PGA alone group also showed incomplete nerve regeneration in the expected RLN area as well as the M group, with very few axon formations (Figure S1), representing the results of wholly no functional recovery of PGA and M group in Figure 2C. Furthermore, transplanted PGA itself were absorbed within four weeks after operation (data not shown). According to the above results, further following morphological analysis was done to concentrate on the HY group. 

**Figure 4:**
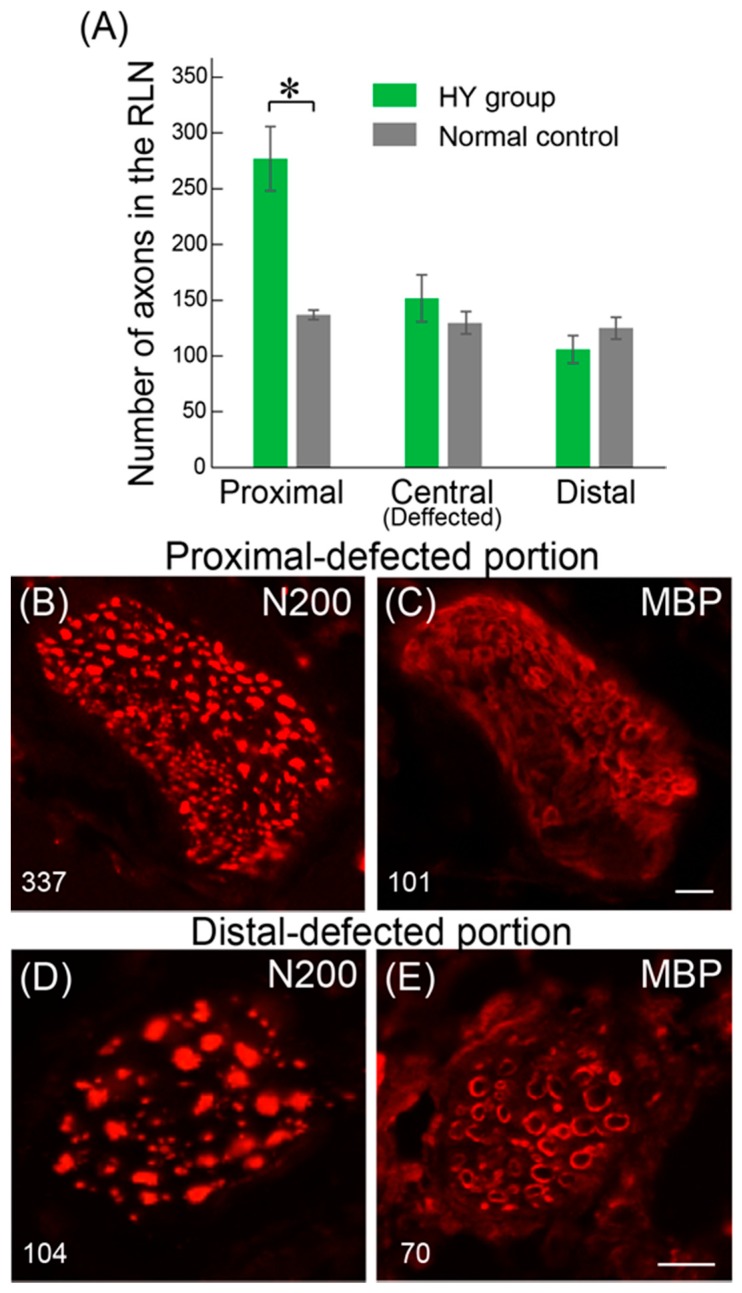
Quantitative analysis of the RLN in the proximal-, central- and distal-portion of nerve-defected area of the HY group. (**A**) The number of axons was gradually decreased following the order of proximal-, central- and distal-portions. The double score is detectable in the proximal-portion, but the score is similar or less in the central and distal-portion in the HY group in comparison to the normal intact control. * *p* < 0.01. Typical photographs of proximal- and distal-potions are shown in (**B**–**E**). Proximal-portion show a 337 axon (N200) count and 101 myelin (MBP) count. The distal-portion shows 104 axons and a myelin count of 70. Note that the proximal-portion shows the existence of aggregation with many small non-myelinated fibers in the left-bottom site ((**B**) proximal-defected portion), but there is no aggregation ((**D**) distal-defected portion). Bars = 50 μm.

The number of regenerated axons was also counted in the pre- (corresponding to Figure 3, Panel 6), central- (Figure 3, Panel 3 or 4), and post-portion (Panel 1) of the defect nerve area, and compared to the normal intact nerve values (Figure 4A). In the pre-portion of the defect nerve area, the number of axons was 277 ± 29, and this number was gradually decreased in the central- (152 ± 21) and in the post-portions (106 ± 12) in the HY group, whereas the normal non-operated control showed 137 ± 4 in pre- and 125 ± 10 in post-portions (Figure 4A). Thus, the HY group showed over double the number of axons as observed in the pre-portion, but similar or less axons than the control in the post-portions. Consequently, the axonal recovery ratio of the post-portion, which is considered the portion nearest to the vocal cord innervation in the RLN, was calculated to be about 85%. The photographs of typical pre- and post-portions of RLN in the HY group are shown in Figure 4B–E. 

Similarly, photographs of typical pre- and post-nerve defected portions are shown in Figure 4B–E. In the pre-defected portion, over double the axon score (337) of the normal control value was confirmed (Figure 4B), but the count of myelinated fibers was 101 (Figure 4C), and the myelination ratio accounted for about 30%. Interestingly, aggregation of a large number of small non-myelinated fibers was observed in the lower left portion of the RLN (Figure 4B,C), and it was obvious that this aggregation largely affected the axon count in this portion. However, the axon count was decreased to 104 (Figure 4D) and the myelin count to 70; thus, the myelination ratio accounted for 67% in the post-portion (Figure 4D,E). Note that the aggregation of small fibers was not observed in this portion. In our pooled data, the myelination ratio of intact RLN in the same portion was about 90%; thus, the recovery ratio of myelinated fibers accounted for 74%.

### 3.4. Differentiation of Engrafted Sk-MSCs

**Figure 5:**
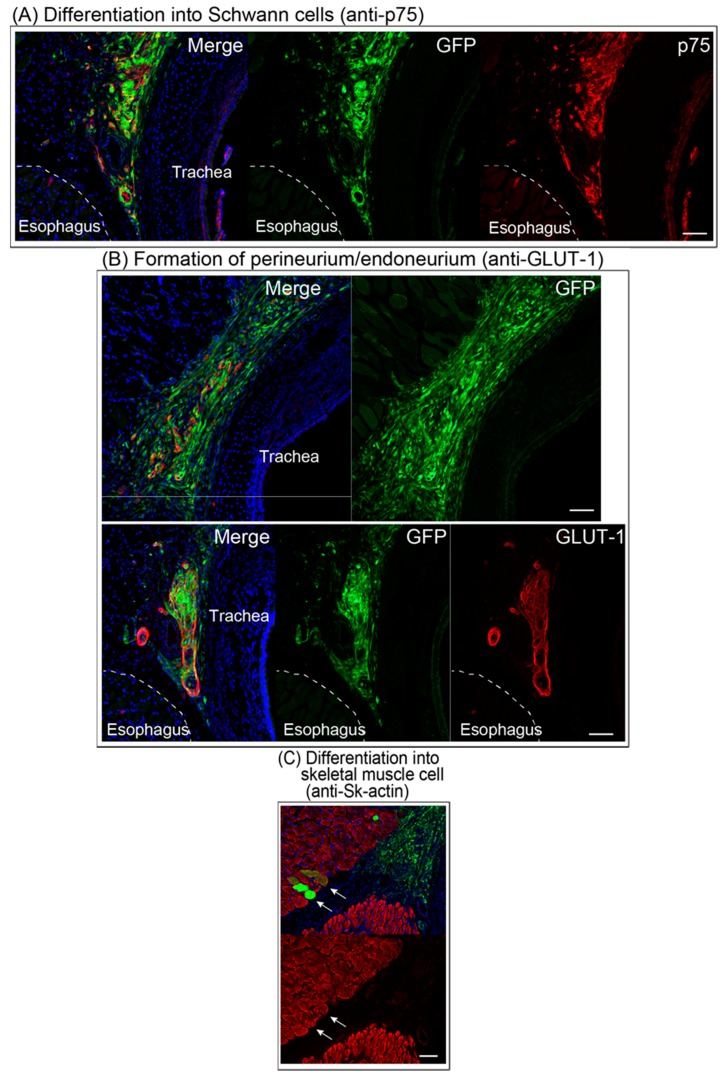

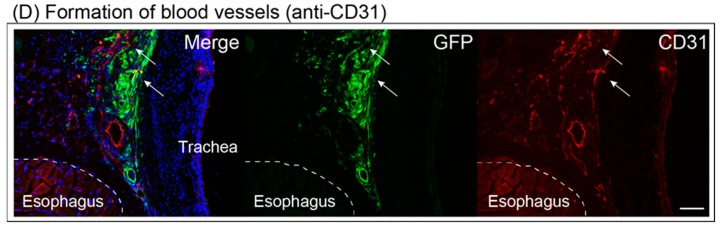
Differentiation of engrafted GFP^+^ cells after eight weeks of transplantation. (**A**) Differentiation into Schwann cells. Anti-p75^+^ (Schwann cell marker, red)/GFP^+^ cells were observed close to the esophagus and trachea, which is the situation of the RLN. (**B**) Formation of perineurium/endoneurium in the RLN situation. Anti-GLUT-1^+^ (marker for the perineurium/endoneurium, red)/GFP^+^ cells also located in the RLN situation forming lots of typical large and small circle-like shapes. (**C**) Typical formation of skeletal muscle cells (anti-skeletal actin^+^ fibers, arrowheads) in the tracheal muscle. (**D**) Formation of blood vessels confirming anti-CD31 in the area of the RLN. Some of these were double positive for CD31 and GFP, showing endothelial cell differentiation. Blue staining in all panels is nuclear staining of DAPI. Bars = 100 μm.

Differentiation of transplanted Sk-MSCs was examined by immunohistochemistry (Figure 5). Transplanted GFP^+^ Sk-MSCs engrafted and closely adhered to the trachea, showing double positive reactions for the Schwann cell marker p75 and GFP (Figure 5A). Thus, transplanted Sk-MSCs differentiated into Schwann cells. Similarly, GFP^+^ and GLUT-1^+^ cells/tissues were also frequently detectable in the cloud of GFP^+^ cells (Figure 5B), showing the differentiation into perineurial/endoneurial cells and/or the formation of perineurium/endoneurium. Differentiation of Sk-MSCs into skeletal muscle cells (fibers) was confirmed by anti-skeletal muscle actin (Figure 5C). Formation of blood vessels was detected by anti-CD31, the endothelial cell marker, and several cells were GFP^+^/CD31^+^ (Figure 5D, arrows), indicating differentiation into endothelial cells. Consequently, transplanted Sk-MSCs exerted capacity for differentiation into Schwann cells, perineurial/endoneurial cells, skeletal muscle cells, and endothelial cells. 

### 3.5. Variations in Axonal Regeneration in the Nerve Defect Central- and Pre-Portions

**Figure 6:**
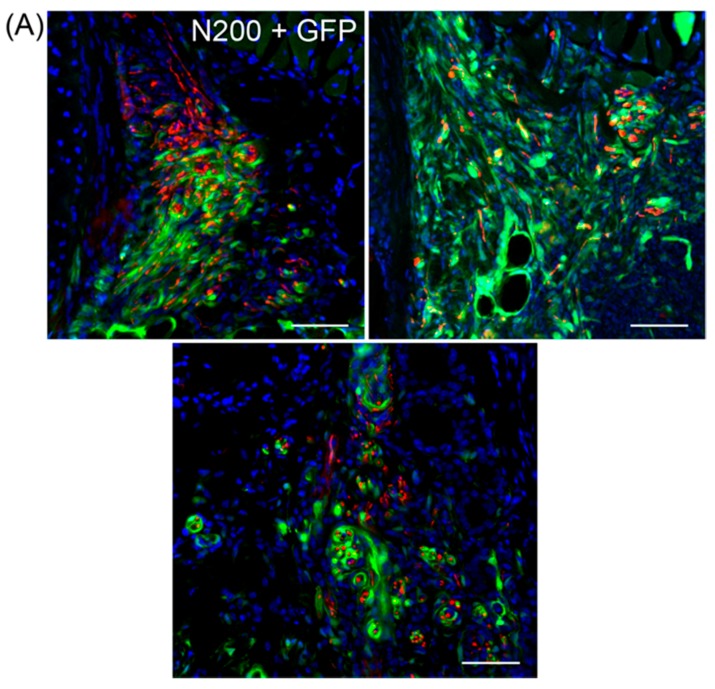

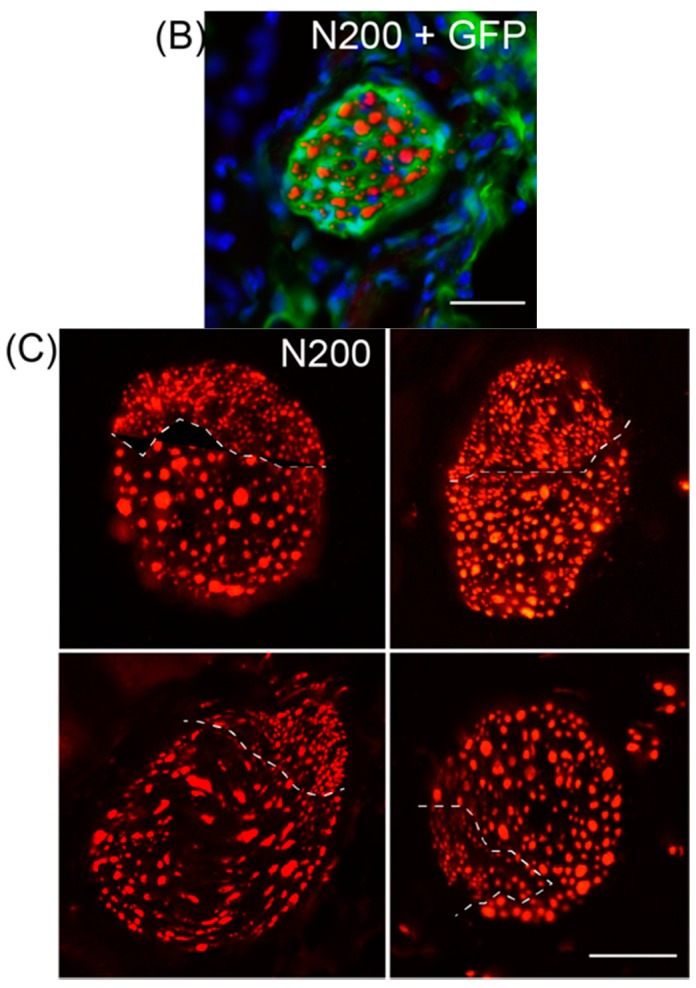
Variations in axonal regeneration in the central-portion (**A** and **B**) and pre-portion (**C**) of the HY group. (**A**) Spreading pattern; and (**B**) concentrate patterns were observed as the variations in the central-portion. A close and consistent relationship between axon (red N200) and GFP^+^ reactions are evident in both patterns. (**C**) Aggregation of many small axons together in one place as sorted by dotted lines in typical four patterns. These are mostly consistent pattern in the pre-portion of the RLN. Blue staining in (**A**,**B**) is nuclear staining of DAPI. (**A**,**C**) Bars = 100 μm; (**B**) Bar = 50 μm.

Variations in axonal regeneration in the central (nerve defected) portion of the HY group are shown in Figure 6A,B. In the nerve-defected portion, extended axons mostly showed the spread pattern to both a large and small extent (Figure 6A), and a concentrated pattern was rarely observed, as shown in Figure 6B. However, this concentrated pattern is a major pattern in the post-portion, which is close to the vocal-cord innervation part, as shown in Figure 3 (HY group). Therefore, combined with the data shown in Figure 4, it was suggested that the nerve fibers once spread in the defected portion, but they were re-concentrated before innervation to the vocal-cord, probably in the distal stump of the RLN. The data clearly reconfirmed the close relationship of transplanted GFP^+^/Sk-MSCs and regenerating axons, as GFP^+^ reactions tightly encircled red (N200^+^) axons (as there were probably Schwann cells and perineurium/endoneurium, Figure 6A).

In contrast, variations in axon distribution in the pre-nerve defected portion are shown in Figure 6C. Axons with a small diameter aggregating in a particular portion (indicated by dotted lines) was observed as a major trend. 

### 3.6. Paracrine Effect of HY Transplantation in Vivo

**Figure 7:**
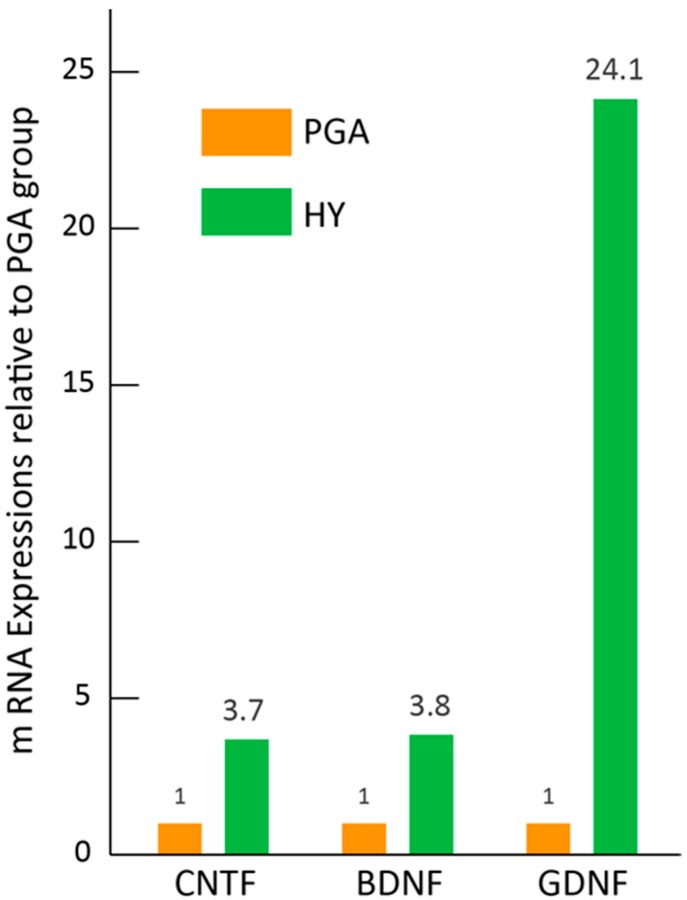
Expression of peripheral nerve growth promotional factor mRNAs in the HY group. Expressions of CNTF, BDNF, and GDNF in the HY group were examined by real-time qPCR relative to the PGA group. The HY group showed higher values of all three factors (3.7–24.1-fold) than the PGA group. Values were obtained as the total value of *n* = 4 samples in each group. This means that the value come from mixed four samples, as the total value (as it is an averaged value) of the group, thus, there are no error bars here.

To confirm the paracrine effects of the HY transplantation, real-time qPCR was performed (Figure 7). The expression of selected nerve growth factor mRNAs in the transplanted solo PGA + medium and PGA + Sk-MSCs after two weeks of transplantation was examined and compared between both groups. The results clearly indicated higher expression of BDNF (3.8-fold), CNTF (3.7-fold), and GDNF (24-fold) in the HY group. Thus, paracrine effects of the HY transplantation, which could contribute to nerve regeneration, were observed. 

## 4. Discussion

Generally, a cell pellet is considered fluid containing cells (cell suspension), and the fluid is strongly affected by the surrounding pressure and/or the capillarity with surface tension, such as in the narrow delta regions in the interspace between the trachea and esophagus. In this scenario, the prevention of transplanted cell diffusion is difficult. This is also the case in the application of other liquid substances such as those containing cytokines, factors, and medicines, because liquid rapidly moves longitudinally along the interspace. The present hybrid cell transplantation system with Sk-MSCs and PGA clearly demonstrated the favorable prevention of transplanted cells/factors diffusion (Figure 1B,C). Similar prevention of cell diffusion has also been reported, as in the case of cell transplantation to the bronchial stump in the pleural cavity, which was considered an unstable condition of cell restriction, as the tissue surface in a free-air space [16]. Therefore, the present hybrid cell transplantation system exerted better restriction on cell diffusion. In contrast, the importance of the prevention of cell diffusion using some kind of scaffold when delivering cells, factors, and/or cytokines onto the surface and/or interspaces of tissues/organs was indicated. Thus, we believe that the present results may be an important first step in the use of this stem cell delivery method. 

In addition to the cell restriction ability of the present hybrid transplantation system, the transplanted/engrafted cells sufficiently differentiated into axon-supportive Schwann cells, perineurial/endoneurial cells, skeletal muscle cells, and endothelial cells, as reported previously [6,8,9]. As a result of this cell differentiation, axons of the growth cone of RLN extended and re-connected to target axons in the distal stump, through the support of Schwann cells, perineurium/endoneurium, and blood vessel networks (Figure 5), and achieved favorable functional recovery (Figure 2), as reported previously [6,7,8,9,10,11,12,13,14,15,16]. In this regard, it is also apparent that this hybrid system does not interrupt any cell differentiation and tissue reconstitution ability of Sk-MSCs. 

In the functional recovery of the damaged peripheral nervous system, restoration of function in the peripheral effector organ is the most important therapeutic goal. In light of this viewpoint, we evaluated vocal-fold movement using trans-oral laryngoscopy (Figure 2 and Table 1), and the HY group showed 80% recovery. To the best of our knowledge, this is a groundbreaking result. For example, in the comparison between bridging a novel PGA tube filled with collagen fibers with a 1 mm gap and the direct sutured control group in the rat RLN transected model, nerve fiber connections were observed macroscopically in both groups, but more clear myelinated fibers and better prevention of laryngeal muscle atrophy were observed in the tube-treatment group at 15 weeks after treatment [17]. However, vocal-fold movement recovery was not observed in either group, and the conduction velocity of the RLN did not differ between the two groups [17]. 

Similarly, for rat transected RLN treatment using a collagen scaffold loaded with laminin and laminin-binding BDNF and GDNF, the treatment group revealed significant improvement (*p* < 0.05) in recovery of vocalization, arytenoid cartilage angles, compound muscle action potentials, and regenerated fiber area in comparison to the autologous nerve grafting group [3]. However, vocal-fold movement was not restored until 12 weeks after treatment [3]. In this report, importantly, it was also demonstrated that the collagen scaffold and laminin alone are not sufficient to promote nerve recovery [3]. The present study also demonstrated poor functional recovery with a tiny axon formation in the solo PGA group (Figure 2 and Figure S1). In contrast, higher expression (3.4–24-fold) of BDNF, CNTF, and GDNF mRNAs, which can promote peripheral nerve regeneration [3,4], was also observed in the HY group (Figure 7). Taken together with the above, the following key points were identified: (1) use of a nerve-bridging substance tended to provide better recovery than direct suture or nerve autograft; (2) the PGA is likely superior to collagen as a substance to bridge the nerve defect; (3) neurotrophic factor application is important for nerve regeneration with the combination of the appropriate scaffold (probably PGA); (4) use of PGA is a better method to deliver the additional nerve growth factors; and (5) the present HY transplantation system maintains all of the above conditions associated with the additional stem cell differentiation into peripheral nerve-supporting cells. Thus, we suggest that the multiple and comprehensive effects of the present HY system may enable 80% recovery of vocal-fold movement. 

Alternately, the most worrisome complication of nerve regeneration is the misdirection of regenerating axons to connect to an inappropriate target [24,25], and this is considered to be frequently observed as a result of a forced mismatch of endoneurial sheaths in the case of end-to-end nerve anastomosis or nerve autografting, and is likely to limit functional recovery [26]. Concerning this point, we considered that the HY group axon count that was over double the score of the normal control at the pre-portion (Figure 4A) may reflect axonal sprouting in the pre-portion growth cone [27,28]. The combined number of axons in the regeneration and degeneration state were included in this score. Therefore, an increase in the number of non-myelinated small fibers, which is seen as an aggregation in Figure 4B,C, may be degenerating and/or degenerated fibers at eight weeks after treatment. This notion is supported by the fact that these small axon aggregations were an almost consistent trend in the pre-portions in the HY group (Figure 6C). Consequently, the increased number of axons in the pre-portion decreased through the nerve-defected central portion, and then, there was concentrated to a similar number to the normal count in the post-portion (Figure 4 and Figure 6). Based on these data, we hypothesized that the proximal stump of the transected nerve increased once the axon number in the growth cone by axonal sprouting during the early stage of regeneration. Then, axon branches extended together toward the target distal axons, and when some of them reached the distal appropriate axons, axonal regeneration/reconnection was completed. Remaining axon branches were gradually demyelinated and degenerated, and may have disappeared finally. The important point is that these selections occurred in the central defected nerve portion, and the transplanted Sk-MSCs contributed to this process biologically and morphologically via the auto and paracrine effects of nerve growth factors. This is the advantageous effect of the present hybrid transplantation system, and we believe that this natural and helpful selection process may have reduced axonal misdirection and accelerated axonal reconnection, resulting in 80% recovery of the vocal-fold movement. This notion is also supported by the randomized controlled clinical studies, which was indicated a better functional recovery can be achieved by intentional tube-bridging with a 3–5-mm long gap between both stumps in comparison to end-to-end nerve suturing [29,30]. This finding, in which the addition of room for nerve re-connection induced better recovery, was also confirmed in the rodent model [25]. Finally, the existence of Sk-MSCs/Sk-SCs, their appropriate isolation/expansion culture method, and their nerve repair ability, have already been confirmed in the mouse and human skeletal muscle [6,7,10]. Therefore, we believe that the present results may be a significant and practical first step towards stem cell transplantation therapy for future thoracic surgery.

## 5. Conclusions

These results show that the stem cell and cytokine delivery system using hybrid transplantation of Sk-MSCs/PGA-felt is a potentially practical and useful approach for the recovery of transected RLN. This system never disturbed the differentiation/reconstitution capacity of co-transplanted Sk-MSCs, and PGA-felt strictly prevented the diffusion of co-transplanted Sk-MSCs, even in the large free space as the plural cavity. More expand in clinical application is expected in the stem cell regenerative medicine. 

## Figures and Tables

**Table 1:** Changes in the distribution ratio of the recovery point criteria of vocal-fold movement in the HY group.

Evaluation	2w (*n* = 6)	4w (*n* = 5)	8w (*n* = 10)
Point 0	100%	20%	0%
Point 1	0%	20%	20%
Point 2	0%	20%	10%
Point 3	0%	40%	70%

## Supplementary Materials

The following are available online at http://www.mdpi.com/2077-0383/7/9/276/s1. Video S1: A video clip of typical vocal-fold movement, rated from 0–3 points in relation to Figure 2 and Table 1. Figure S1 presents the formation with very few axons in the RLN defected portion of the PGA group.
