# Supplementary material for: Regeneration of Transected Recurrent Laryngeal Nerve Using Hybrid-Transplantation of Skeletal Muscle-Derived Stem Cells and Bioabsorbable Scaffold
Source: J Clin Med. 2018 Sep 12;7(9):276. doi: 10.3390/jcm7090276 (PMC6162854; doi:10.3390/jcm7090276)
Supplement: Supplementary file 1 [file jcm-07-00276-s001.zip › jcm-350201-SI.pdf]

Journal of
Clinical Medicine

MDPI

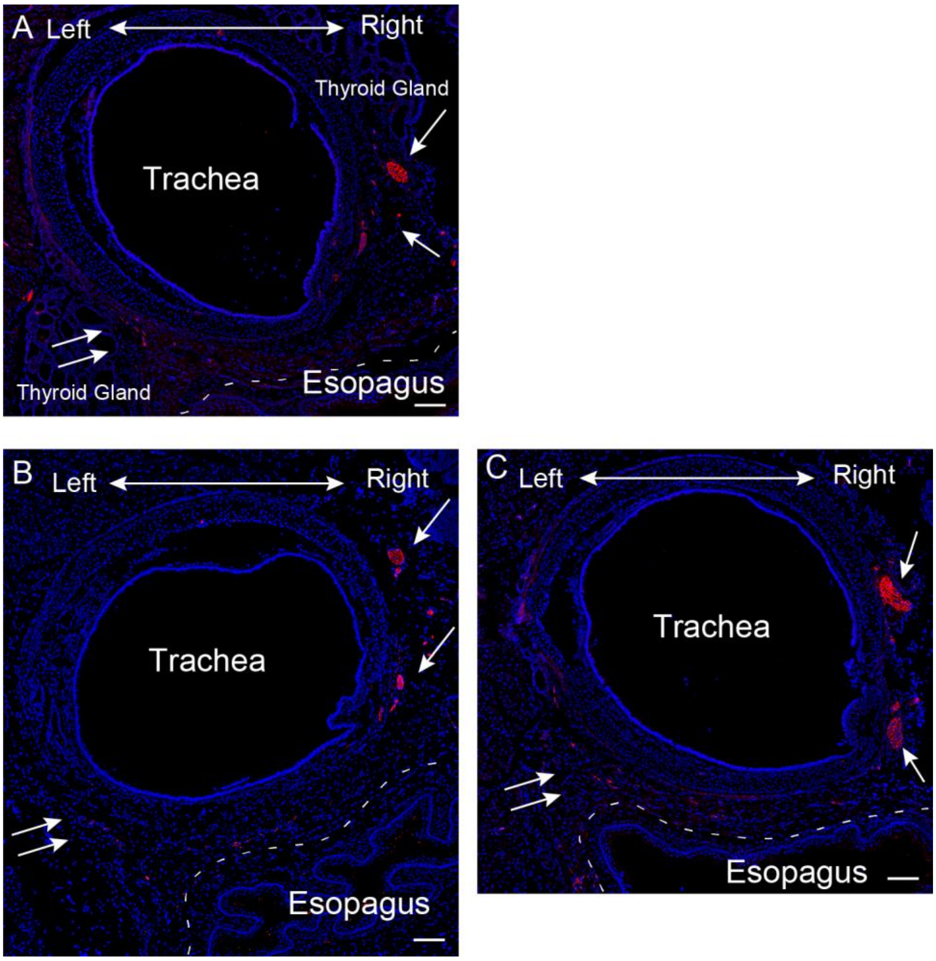

Supplemental Figure S1. A very few axon formation (stained with N200) of the PGA group in the RLN defected portion. There are typical photographs taken from three different animals. A: this corresponds to the situation of panel 1 in Figure 3; M group. B: this corresponds to the situation of panel 3, C: this corresponds to panel 4. Arrows indicate the expected situation of the RLN both in the right and left side. These data consistently represent the results of functional recovery in Figure 2. Blue staining=nuclear staining with DAPI. Bars=100 μm.
